# Frequency-specific alterations of the resting-state BOLD signals in nocturnal enuresis: an fMRI Study

**DOI:** 10.1038/s41598-021-90546-3

**Published:** 2021-06-08

**Authors:** Xiangyu Zheng, Jiawei Sun, Yating Lv, Mengxing Wang, Xiaoxia Du, Xize Jia, Jun Ma

**Affiliations:** 1 Department of Developmental and Behavioral Pediatrics, Shanghai Children’s Medical Center, School of Medicine, Shanghai Jiao Tong University, 1678 Dong-Fang Road, Shanghai, 200127 China; 2 School of Information and Electronics Technology, Jiamusi University, Jiamusi, Heilongjiang China; 3 Institute of Psychological Sciences, Hangzhou Normal University, Hangzhou, 311121 Zhejiang China; 4 Zhejiang Key Laboratory for Research in Assessment of Cognitive Impairments, Hangzhou, 311121 China; 5 College of Medical Imaging, Shanghai University of Medicine & Health Sciences, Shanghai, 201318 China; 6 Department of Physics, Shanghai Key Laboratory of Magnetic Resonance, East China Normal University, 3663 North Zhong-Shan Road, Shanghai, 200062 China

**Keywords:** Neuroscience, Urology

## Abstract

Resting state functional magnetic resonance imaging studies of nocturnal enuresis have focused primarily on regional metrics in the blood oxygen level dependent (BOLD) signal ranging from 0.01 to 0.08 Hz. However, it remains unclear how local metrics show in sub-frequency band. 129 children with nocturnal enuresis (NE) and 37 healthy controls were included in this study. The patients were diagnosed by the pediatricians in Shanghai Children’s Medical Center affiliated to Shanghai Jiao Tong University School of Medicine, according to the criteria from International Children's Continence Society (ICCS). Questionnaires were used to evaluate the symptoms of enuresis and completed by the participants. In this study, fALFF, ReHo and PerAF were calculated within five different frequency bands: typical band (0.01–0.08 Hz), slow-5 (0.01–0.027 Hz), slow-4 (0.027–0.073 Hz), slow-3 (0.073–0.198 Hz), and slow-2 (0.198–0.25 Hz). In the typical band, ReHo increased in the left insula and the right thalamus, while fALFF decreased in the right insula in children with NE. Besides, PerAF was increased in the right middle temporal gyrus in these children. The results regarding ReHo, fALFF and PerAF in the typical band was similar to those in slow-5 band, respectively. A correlation was found between the PerAF value of the right middle temporal gyrus and scores of the urinary intention-related wakefulness. Results in other bands were either negative or in white matter. NE children might have abnormal intrinsic neural oscillations mainly on slow-5 bands.

## Introduction

Nocturnal enuresis (NE) is defined as repeatedly urinating in clothes or bed that occurs only during sleep in an individual who has reached a developmental age when urinary continence is ordinarily expected at 5 years old^1,2^, with a prevalence of around 5% in children between 5 and 12 years old^3^. Children who suffer from enuresis often experience emotional distress and have low self-esteem^4,5^. Previous reports have shown that maturational delay of the central nervous system is an important factor in the pathogenesis of NE^6^. Thus, the characterization of alterations in brain function in NE may provide an insight into understanding enuresis pathophysiology.

Resting-state functional magnetic resonance imaging (rs-fMRI) is a non-invasive technique and has been widely employed to investigate brain function^7,8^. The RS-fMRI bold signal includes the magnetic field information resulted from the changes of oxygen and hypoxia in hemoglobin supplied to neuron blood^9^. Previous research findings suggested that bold signals are significantly correlated with field potentials reflecting electrical signals in the neural networks^10^. In recent years, a variety of metrics has been proposed in the field of rs-fMRI that allow us to understand the spontaneous neural activity of the subject's brain from various perspectives.

Amplitudes of low-frequency fluctuations (ALFF) and Regional homogeneity (ReHo) are two metrics particularly widely used for uncovering local spontaneous neural activity in the brain and have been used in thousands of articles^11,12^. ReHo is used to analyze spontaneous synchronization of local activity in the brain^13^, whereas ALFF is used to analyze the amplitude of single voxel low frequency oscillations (amplitude)^14^. However, ALFF was previously found susceptible to physiological noise such as respiratory and heartbeat. Therefore, an improved ALFF algorithm named fractional amplitude of low-frequency fluctuation (fALFF) was proposed and able to be used to suppress noise and to provide a better one-sample t-test patten than ALFF^15^. The signal power, fALFF, normalized by the power of the whole spectrum, effectively suppresses noise, though reduces the test–retest reliability^15,16^. PerAF, as a latest voxel-level amplitude metric, has the better retest reliability, both intra-machine and inter-machine than ALFF and fALFF^16,17^. Therefore, ReHo, fALFF and PerAF were both applied in this study.

In recent years, ReHo and fALFF have been widely used in the study of various types of brain disorders. The amplitudes of low frequency fluctuations and local synchronization in the 0.01–0.08 Hz frequency band in the patients with many neuropsychiatric disorders, e.g., depression^18,19^, schizophrenia^20,21^, Alzheimer's disease^22,23^ and Attention Deficit–Hyperactivity Disorder (ADHD)^24,25^ have been found distinguishable from those in the normal controls. As most patients with enuresis are children, it is relatively difficult to collect rs-fMRI data. To our acknowledge, only a few articles focused on local spontaneous activity in NE children^6,26,27^, suggesting abnormalities in left inferior parietal lobule, left inferior frontal gyrus and left medial orbital superior frontal gyrus in the 0.01–0.08 Hz range in NE children compared to healthy controls (HCs).

In recent years, increasing research interests has been on the experiments with non 0.01–0.08 Hz (non-traditional frequency bands)^28,29^. Zuo et al. firstly attempted to divide the rs-fMRI spontaneous signal into four sub-bands [slow-5 (0.01- 0.027 Hz), slow-4 (0.027–0.073 Hz), slow-3 (0.073–0.198 Hz), and slow-2 (0.198–0.25 Hz)]^30^. The signals of gray matter related oscillatory amplitudes were found in slow-5 and slow-4 ranges, while that of white matter signals and high-frequency physiological noises mainly in slow-3 and slow-2. Many studies have revealed frequency-dependent abnormalities in neurological and psychiatry diseases including autism spectrum disorder^31^, schizophrenia^32^, and depression^29^. Given limited findings in the studies related with the band 0.01–0.08 Hz, further information may be provided sub-bands (e.g. slow-4, slow-5) otherwise.

Based on previous study, we hypothesized that there were frequency-dependent abnormalities in children with NE. Therefore, to verify the results of previous studies and examine the frequency specificity of brain activity in children with enuresis, we compared the spontaneous activities between children and HCs in the typical frequency range and four different low-frequency bands.

Besides, many studies indicated that children with NE had increased arousal level than health controls, unable to wake in sleep even though the bladder was filled^33^. Abnormal urinary intention-related wakefulness played an important role in the occurrence of NE^34^. However, there were few studies exploring the relationship between the degree of urinary intention-related wakefulness and local brain abnormalities in children with NE. Therefore, correlation analysis was performed between index of spontaneous activity of local brain regions and scores on urinary intention-related wakefulness in different bands to detect frequency-dependent abnormalities in NE children.

## Results

Compared with that of previous fMRI studies on nocturnal enuresis, the sample size of this research was the largest^6,27,35–37^. There were 129 NE children and 37 HCs enrolled in this study. Necessary exclusion was made due to various reasons. For example, 5 subjects with the head movement larger than 3.0 mm of translation or 3.0° of rotation were ruled out and 8 subjects due to poor registrations. More details could be seen in Methods.

There was no significant difference in age between NE and HCs (t = 1.118, P = 0.266). However, the gender difference between the two groups was statistically significant (χ^2^ = 7.807, P = 0.005), as displayed in Table 1. Covariance regression was conducted in our study as reduction in the difference caused by the group gender was widely reported in previous studies^38,39^. Thus, gender was considered as a covariate in the regression model.

**Table 1:** Demographic data of the study participants.

	NE participants (n = 76)	Control participants (n = 30)	Chi-square or t value	P value
Age	9.27(± 1.760)	9.68(± 1.601)	1.118	0.266
**Gender**			7.807	0.005
Boys	57(75%)	14(46.7%)		
Girls	19(25%)	16(55.3%)		
The frequency of NE	76	30	55.378*	< 0.01
No enuresis	1	30		
1–3 times a month	10	0		
Once a week	3	0		
Twice a week	5	0		
Three times a week	8	0		
Four times a week	6	0		
Five times a week	0	0		
Six times a week	0	0		
Once per night	25	0		
Twice per night	15	0		
Three times per night	3	0		
Four times per night	0	0		
Five times per night	0	0		
Above five times per night	0	0		
Urinary intention-related wakefulness	76	30	87.233*	< 0.01
No enuresis	1	30		
Wake-up after urinating less than half of urine volume	3	0		
Wake-up after urinating more than half of urine volume	3	0		
Wake-up after empty the bladder	16	0		
Inability to wake up after emptying the bladder	53	0		

*The result came from Mantel–Haenszel test.

**Figure 1:**
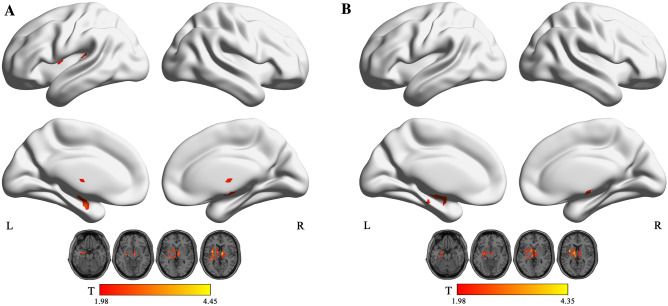
Brain regions with abnormal ReHo in typical low-frequency range and at slow-5 band in NE children. The results were corrected by GRF (voxel P < 0.05, cluster P < 0.05). More details of these regions were described in Table 2. (**A**) Left insula and right thalamus with increased ReHo in typical low-frequency range (0.01–0.08 Hz). **B** Left thalamus with increased ReHo in slow-5band (0.01-0.027 Hz).

**Figure 2:**
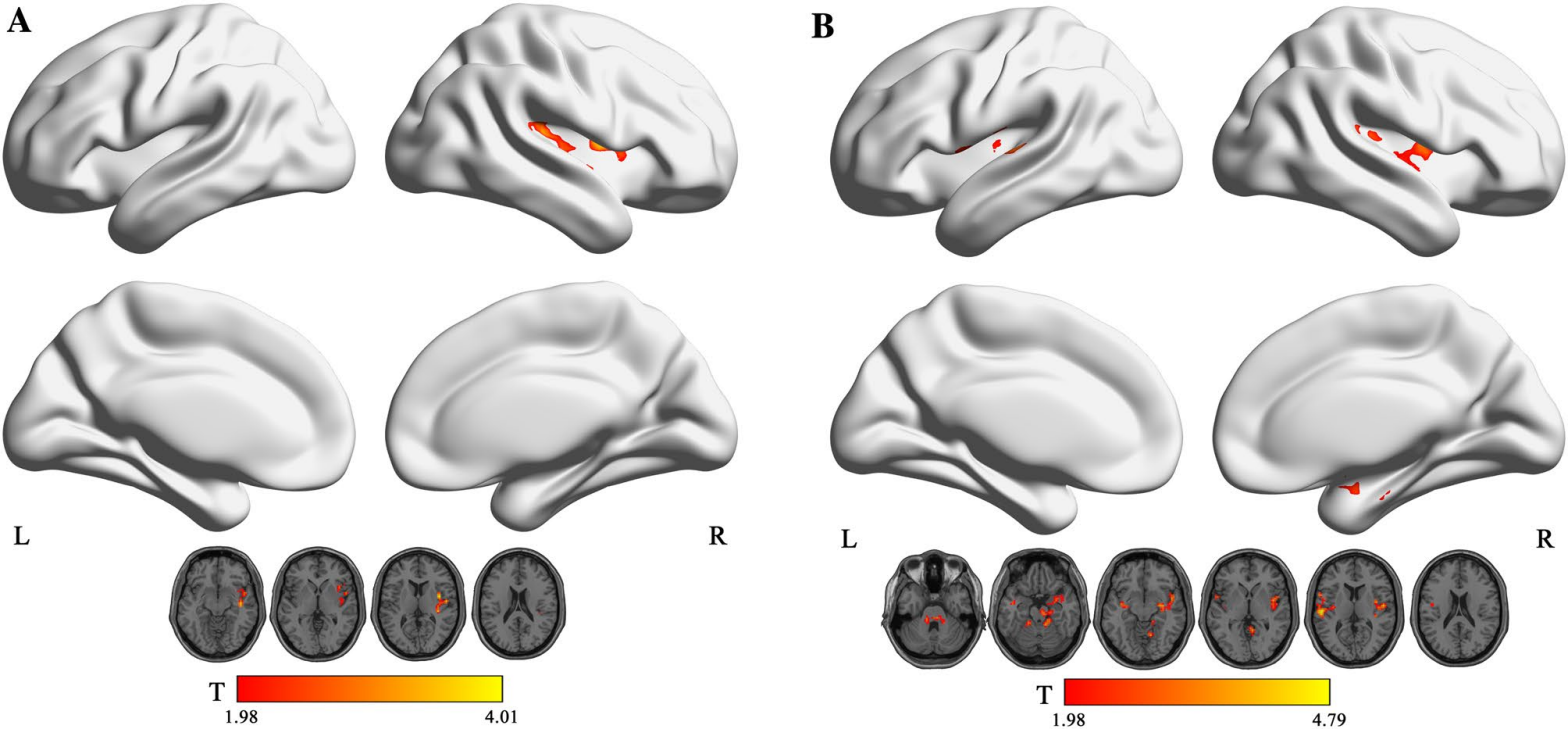
Brain regions with abnormal fALFF in typical low-frequency range and at slow-5 band in NE children. The results were corrected by GRF (voxel P < 0.05, cluster P < 0.05). More details of these regions were described in Table 3. (**A**) Left insula with increased fALFF in typical low-frequency range (0.01–0.08 Hz). (**B**) Superior cerebellum, right rolandic operculum, superior temporal gyrus with increased fALFF in slow-5 band (0.01–0.027 Hz).

**Figure 3:**
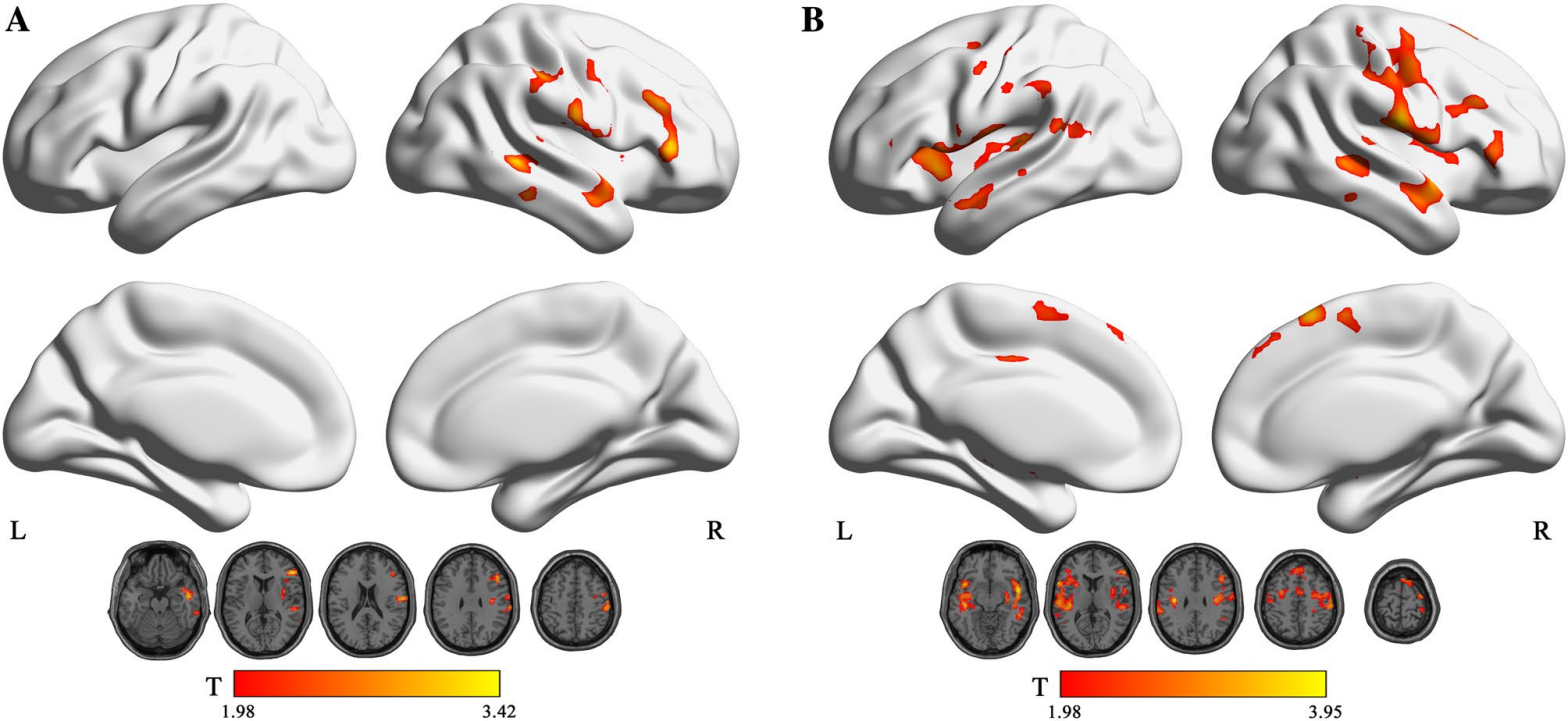
Brain regions with abnormal PerAF in typical low-frequency range and at slow-5 band in NE children. The results were corrected by GRF (voxel P < 0.05, cluster P < 0.05). More details of these regions were described in Table 4. (**A**) Right middle temporal gyrus with increased PerAF in typical low-frequency range (0.01–0.08 Hz). (**B**) Left insula, right temporal pole: middle temporal gyrus with increased PerAF in slow-5 band (0.01–0.027 Hz).

**Figure 4:**
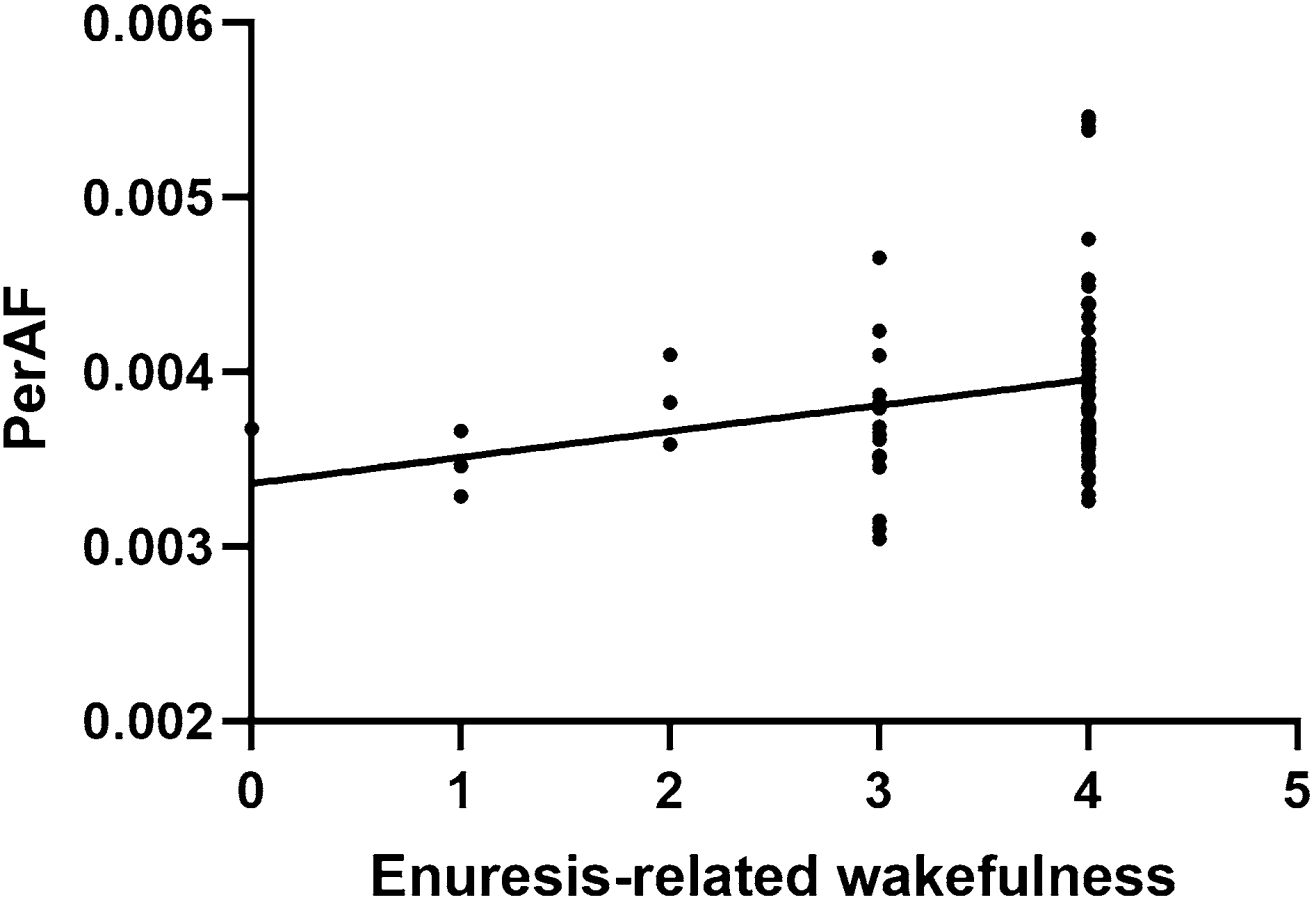
PerAF in middle temporal gyrus was positively associated with scores of urinary intention-related wakefulness.

The results showed that in the typical band, ReHo increased in the left insula and the right thalamus in NE (voxel P < 0.05, cluster P < 0.05; Fig. 1A, Table 2). In addition, fALFF increased in the right insula in NE (voxel P < 0.05, cluster P < 0.05; Fig. 2A, Table 3). Moreover, PerAF increased in the right middle temporal gyrus in NE (voxel P < 0.05, cluster P < 0.05; Fig. 3A, Table 4). In slow5-band, ReHo increased in the thalamus in NE (voxel P < 0.05, cluster P < 0.05; Fig. 1B, Table 2), and fALFF increased in the superior cerebellum, superior temporal gyrus in NE (voxel P < 0.05, cluster P < 0.05; Fig. 2B, Table 3). The results in other bands were either negative or in white matter, where previously most BOLD signals were usually considered as noise and the white matter signal in the regression model. PerAF increased in left insula and temporal pole: superior temporal gyrus (voxel P < 0.05, cluster P < 0.05; Fig. 3B, Table 4). Results in other bands were either negative or in white matter (more details could be seen in supplementary materials). PerAF in the right middle temporal gyrus in typical band was positively associated with the scores of urinary intention-related wakefulness (r = 0.252; P = 0.0281; Fig. 4).

**Table 2:** Comparisons of Regional Homogeneity between groups in typical low frequency range (0.01–0.08 Hz) and slow-5 frequency bands (GRF Corrected, Overall P < 0.05). All the coordinates are denoted by Montreal Neurological Institute (MNI) space coordinates.

Anatomical label	BA	Number of voxels	Peak MNI coordinates [x,y,z]	Peak T-value
**Typical band**				
Left insula		378	− 330, 9	4.4449
Right thalamus		404	9, − 9, 9	4.2923
**Slow-5**				
Left thalamus		820	− 6, − 21, 6	4.3479

**Table 3:** Comparisons of fractional Amplitude of Low Frequency Fluctuation between groups in typical low frequency range (0.01–0.08 Hz) and slow-5 frequency bands (GRF Corrected, Overall P < 0.05). All the coordinates are denoted by Montreal Neurological Institute (MNI) space coordinates.

Anatomical label	BA	Number of voxels	Peak MNI coordinates [x,y,z]	Peak T-value
**Typical band**				
Right Insula	48	291	51, 6, − 3	4.0045
**Slow-5**				
Superior cerebellum		335	− 12, − 36, − 27	3.7339
Right rolandic operculum	43	374	51, − 9, 9	4.2472
Superior temporal gyrus	41	290	− 57, − 18, 9	4.7832

**Table 4:** Comparisons of Percentage Amplitude Fluctuation between groups in typical low frequency range (0.01–0.08 Hz) and slow-5 frequency bands (GRF Corrected, Overall P < 0.05). All the coordinates are denoted by Montreal Neurological Institute (MNI) space coordinates.

Anatomical label	BA	Number of voxels	Peak MNI coordinates [x,y,z]	Peak T-value
**Typical band**				
Right middle temporal gyrus		1353	54, − 42, − 3	3.411
**Slow-5**				
Left insula		2557	− 42, 12, − 6	3.8888
Right temporal pole: middle temporal gyrus		2390	51, 3, − 6	3.9484

## Discussion

In the present study, we analyzed the ReHo, fALFF and PerAF of children with enuresis in four sub-frequency bands and typical band, respectively. In typical band, ReHo increased in the left insula and the right thalamus in NE, and fALFF was increased in the right insula in NE. Moreover, PerAF was increased in the right middle temporal gyrus in NE. In slow-5 band, ReHo was increased in the thalamus in NE, and fALFF was increased in the superior cerebellum, superior temporal gyrus in NE. PerAF was increased in left insula and temporal pole: superior temporal gyrus. Results in other bands were either negative or in white matter, where previously most BOLD signals were usually considered to be noise and would regress to the white matter signal^40,41^.

The unusual fALFF and PerAF in NE existed in slow-5 band, while fALFF in slow-2 were mainly in white matter and appeared negative in other bands. Similarly, PerAF in NE children was negative in the other bands. Since the oscillations in different frequency bands reflect the characteristics of different oscillators^30^, the analysis in a specific sub-band was able to suggest the spontaneous activity more specifically after removing the interfering signals in other frequency bands^42^. Although oscillations in brain activity was mainly regarded as electrophysiological signals, it also has been used in fMRI study^30,32^. Based on the specificity of brain activity in NE children, frequency-specific alteration might contribute to classification of the diagnosis of NE, which has been used for many disease classification^31,43^.

In our study, increased fALFF was presented in the superior cerebellum of NE children in slow-5. Patients with cerebellar lesions had a hyperreflexia to urinate, possibly indicating that the cerebellum had an inhibitory effect on voiding^44^. However, there were few studies verifying this function in NE children. In other aspects, Yu et al. found the memory/caution factor correlated with the connectivity between cerebellum and middle frontal gyrus in children with enuresis^45^. In addition, structural differences in the cerebellum were also associated with attention/memory deficits in enuretic children through voxel-based morphometry (VBM) methodology^46^. The functional changes of cerebellum in NE children might be related to micturition function and congnitive function as well.

Both PerAF and fALFF increased in temporal lobe in NE in solw-5. Stasa D. et al. reported that temporal lobe was activated obviously during infusion of fluid in the bladder^47^. Lingna Zhao et al. also found that the functional connectivity between the temporal gyrus and the insula was enhanced when the bladder was full^48^. Detrusor overactivity was a feature of NE children^49^. The functional changes of the temporal lobe in NE children might be related to urinary storage dysfunctions. In the present study, PerAF value of the left insula was increased in children with NE in slow-5, suggesting that the spontaneous activity increased in insula after we calculated the percentage of BOLD fluctuations relative to the mean BOLD signal intensity for each time point and averaging across the whole time series. In previous study observed was that the insular activation increased with bladder filling so as to stir a desire to void^50^. The increased spontaneous overactivity and local synchronization of the insula might be an underlying mechanism for the decreased consciousness of controlling urine in NE children^51^. Insula was also found related with awareness, attention and motor response inhibition^36,52^. Consequently, functional changes in the insula might be a factor in abnormalities in the perception of urination and cognitive function in NE.

Furthermore, we also found that unusual ReHo in NE was linked with slow-5 band, while the results in slow-2 and slow-3 were mainly in white matter and negative in other bands . Oscillatory synchrony in specific frequencies were found to be related to particular cells and associated circuits and enabled the brain to be flexible so as to gear to behavioral demands^53^. Apparently, more studies were required to locate the particular oscillators in NE children’s brains. In the present study, higher ReHo values were presented in left thalamus among NE children, indicating that the left thalamus was a possible nidus with a possible increase in local potential^54^. Previous research on brains of NE children suggested not only structural but also functional changes in the thalamus. Moreover, Lei, D. et al. reported that enuretic children showed a decrease in fractional anisotropy (FA) while an increase in mean diffusivity (MD) in the thalamus, indicating developmental delay in thalamus^55^. In addition, the NE group showed remarkably decreased functional connectivity between thalamus and left medial superior frontal gyrus^56^. Furthermore, varying intra thalamic functional connectivity was found in many parts of the thalamus, during light NREM sleep^57^. Besides, Yu, B. et al. reported that longer light NREM sleep and higher awakening index were found related to the changed activity in thalamus^57^. Therefore, the developmental delay in the thalamus might lead to a compensatory increase in the functional connectivity between the voxels within it, which manifests itself macroscopically as abnormal functional connectivity in various parts of the interior thalamus^55,57^. The increased activity of the thalamus might block the conduction of signals in the brain network. As a result, abnormal connections to other brain areas occurred^56^ and eventually contributed to abnormal higher awakening index^57^.

In sum, despite the hypothetical brain regions for enuresis in this study, they were replicated through different metrics, e.g., fALFF and PerAF in different low-frequency band. To our best knowledge, this was the first time in children with NE and described brain abnormalities in terms of signal oscillations, whereas ReHo that did in terms of local synchrony. Functional connectivity^3,57^ described brain abnormalities in terms of loops and connections between brain regions. The importance of spontaneous activity within different frequency band in NE children was highlighted in our study, which might help explore more features of brain activities in NE children. Besides, we found that the right middle temporal gyrus was positively associated with degree of urinary intention-related wakefulness in typical band. Thus, the temporal lobe might be a significant brain area and call for further research.

## Methods

### Subjects

129 children with NE and 37 healthy controls were included in this investigation. The patients were diagnosed by pediatricians in Shanghai Children’s Medical Center affiliated to Shanghai Jiao Tong University School of Medicine, according to the criteria from International Children's Continence Society (ICCS). Nocturnal enuresis refers to intermittent incontinence while sleeping in children at least 5 years old. The exclusion criteria were: (1) any other neurological or psychiatric disorders (e.g., attention-deficit/hyperactivity disorder and autism); (2) metal implants; (3) claustrophobia; (4) IQ test scores lower than 75 (Wechsler Intelligence Scale for Children-Revised); (5) left-handedness; (6) images with inaccuracy registration; (7) head movement larger than 3.0 mm of translation or 3.0° of rotation were excluded. Healthy volunteers, recruited using advertisements, had not wet the bed since 5 years old.

There were 129 NE children from outpatient clinics and 37 healthy controls enrolled for this study from April 2016-August 2017. After ruling out those who met at least one of the exclusion criteria, eventually 76 children with NE and 30 normal children participated in this study. All of the exclusions were objective and indispensable, shown as follows:

1. Five enuretic children with the head movement larger than 3.0 mm of translation or 3.0° of rotation were excluded, because their head motion had been shown to have systematic effects on fMRI data. According to previous studies, in order to mitigate the effect of head movement, head movement larger than 3.0 mm of translation or 3.0° of rotation had been routinely removed during fMRI.
2. One subject in NE group did not meet the criteria, due to wetting bed once per year. According to the criteria from International Children's Continence Society (ICCS), nocturnal enuresis is defined as intermittent incontinence at least once a month and lasting for more than 3 months while sleeping, after the age of 5 years.
3. One NE child was left-handed and thus excluded in this study. In fMRI studies, the identical brain regions were structurally different between left-handed and right-handed people^58^. In the task status, different brain regions were activated between the left-handed and right-handed^59^. Therefore, left-handedness and right-handedness needed to be considered in the studies regarding fMRI. Generally, the subjects in previous studies were right-handed, while the left-handed excluded, representing the majority of the populations.
4. Three subjects were uncooperative with only structures scanned but no functional images obtained. Demographic data of forty NE children were not provided due to confidentiality.
5. In addition, we found that one NE child had unclear T1 images and one NE child and eight healthy participants had incomplete registration.. Consequently, they were excluded from the study.

All the participants were interviewed and filled out a questionnaire that required demographic information and clinical history. In this questionnaire, the frequency of NE (0 for no enuresis; 1 for 1–3 times a month; 2 for once a week; 3 for twice a week; 4 for three times a week; 5 for four times a week; 6 for five times a week; 7 for six times a week; 8 for once per day; 9 for twice per night; 10 for three times per night; 11 for four times per night; 12 for five times per night; 13 for above five times per night) and urinary intention-related wakefulness (0 for no enuresis; 1 for wake-up after urinating less than half of urine volume; 2 for wake-up after urinating more than half of urine volume; 3 for wake-up after emptying the bladder; 4 for inability to wake up after emptying the bladder) were collected. (More details could be found in supplementary materials).

The protocol for this research had been approved by the Ethics Committees of Shanghai Children's Medical Center and was consistent with the provisions of the Declaration of Helsinki and relevant policies in China. All of the participants and their caregivers provided written informed consent before the investigation. Besides, they were informed that the whole survey involvement would be confidential and voluntary and reassured about their right to withdraw from the survey at any time.

### Data collection

The rs-fMRI scanning parameters were repetition time (TR) = 2000 ms; echo time (TE) = 30 ms; flip angle = 90°; FOV = 22 × 22 cm^2^; acquisition matrix = 64 × 64; voxel size = 3.4 × 3.4 × 3 mm^3^; slice number = 32, volume number = 210, scan time = 420 s. T1- weighted image acquisition for all individuals, the imagining parameters are: TR = 440 ms; TE = 2.46 ms; flip angle = 90°; acquisition matrix = 256 × 320; FOV = 22 × 22 cm^2^. During the scan, the subjects were instructed to keep eyes closed.

### Data preprocessing

Preprocessing of resting-state functional data was performed using the DPABI toolbox^60^ in MATLAB R2017b (MathWorks, Natick, Mass). The first 10 time points of each functional time series were discarded for magnetization equilibrium and participant adaptation. Slice timing correction and realignment were applied to the remaining volumes. Afterward, the corrected functional images were spatially normalized to the standard Montreal Neurological Institute (MNI) space with a resampled voxel size of 3 × 3 × 3 mm^3^ by using unified segmentation to the T1 image. 5 subjects with the head movement larger than 3.0 mm of translation or 3.0° of rotation and 8 subjects with poor registrations were ruled out from the study. Linear trends within the time series were removed after spatial normalization. Then, regression models were used to to remove variances regarding head motion parameters from the time series of each voxel^61^. The mean value of the time series of each voxel was added back in this step. Preprocessed functional data were band-pass filtered (The fALFF was not filtered).

### Data analysis

Voxel-wise whole-brain analytic metrics were applied to further data analysis. In this study, fALFF, ReHo and PerAF were calculated using RESTplus^62^ within five different frequency bands: typical band (0.01–0.08 Hz), slow-5 band (0.010–0.027 Hz), slow-4 (0.027–0.073 Hz), slow-3 (0.073–0.198 Hz), and slow-2 (0.198–0.25 Hz). Each metric except PerAF of each voxel was then divided by the global mean metric value of each individual for standardization purposes. Finally, the data were smoothed with a Gaussian kernel of 4 mm full width at half-maximum (FWHM).

### Statistical analysis

To compare the fALFF, ReHo and PerAF maps between HCs and patients with NE respectively, two-sample t-tests were performed within the five different frequency bands mentioned above to identify the regions with significant differences. Gender was treated as covariates during the group comparisons to minimize their potential effect on our results. The resultant T-maps were corrected using the Gaussian random field (GRF) theory (voxel P < 0.05, cluster P < 0.05). Pearson correlation analysis was performed with clinical/physiological/biochemical characteristics of the patients (including frequency of nocturnal enuresis, urinary intention-related wakefulness). All the statistical analysis was performed using DPABI^60^.

## Supplementary Information

Supplementary Information.
