# Supplementary material for: Frequency-specific alterations of the resting-state BOLD signals in nocturnal enuresis: an fMRI Study
Source: Sci Rep. 2021 Jun 8;11:12042. doi: 10.1038/s41598-021-90546-3 (PMC8187680; doi:10.1038/s41598-021-90546-3)
Supplement: Supplementary file 1 — Supplementary Information. [file 41598_2021_90546_MOESM1_ESM.docx]

**Frequency-specific alterations of the resting-state BOLD signals in Nocturnal Enuresis: An fMRI Study**

Xiangyu Zheng ^a^, Jiawei Sun ^b^, Yating Lv ^c,d^, Mengxing Wang ^e^, Xiaoxia Du ^f^, Xize Jia ^c,d*^, Jun Ma ^a*^

^a^ Department of Developmental and Behavioral Pediatrics, Shanghai Children’s Medical Center, School of Medicine, Shanghai Jiao Tong University, 1678 Dong-Fang Road, 200127 Shanghai, China

^b^ School of Information and Electronics Technology, Jiamusi University, Jiamusi, Heilongjiang, China

^c^ Institute of Psychological Sciences, Hangzhou Normal University, Hangzhou, 311121 Zhejiang, China

^d^ Zhejiang Key Laboratory for Research in Assessment of Cognitive Impairments, 311121 Hangzhou, China

^e^ College of Medical Imaging, Shanghai University of Medicine & Health Sciences, 201318, Shanghai, China

^f^ Department of Physics, Shanghai Key Laboratory of Magnetic Resonance, East China Normal University, 3663 North Zhong-Shan Road, 200062 Shanghai, China

Xiangyu Zheng and Jiawei Sun contributed equally to this work.

Correspondence and requests for materials should be addressed to Jun Ma. (email: majun@shsmu.edu.cn); Xize Jia (email: jiaxize@hznu.edu.cn)

**Questionnaire for children with nocturnal enuresis about urinary intention-related wakefulness**

This questionnaire is designed to capture the arousal degree of children with enuresis. Parents are asked to complete it voluntarily and to note the following points.

(1) The questionnaire is based on the child's behavior over the past three months

(2) The child's natural state without outside intervention

Your privacy is fully protected and if you have any questions about this questionnaire, please ask the researcher.

1. Name: ___________________

2. Gender: __________________

3. Date of birth: ______________

4. The frequency of bedwetting based on last three month, without waked by others. _______

A. for not enuresis B. for 1-3 times a month

C. for once a week D. for twice a week;

E. for three times a week F. for four times a week;

G. for five times a week H. for six times a week;

I. for once per night J. for twice per night;

K. for three times per night L. for four time per night;

M. for five time per night N. for above five times per night

5. When the child sleeps at night, can he wake up after receiving urinary stimulation（based on last three month, without waked by others）? ______

A. not enuresis

B. wake up after urinating little

C. wake up after urinating more

D. wake up after empty the bladder

E. inability to wake up after empty the bladder


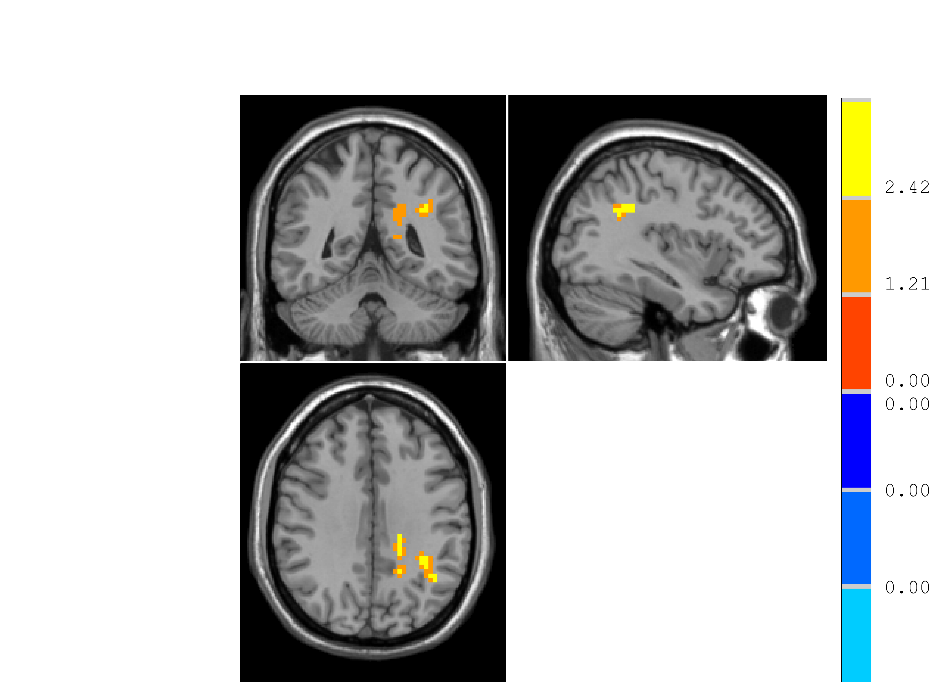


Fig1. Brain regions with abnormal fALFF in typical low-frequency range and at slow-2 band in NE. The results were corrected by GRF (voxel P < 0.05, cluster P< 0.05).


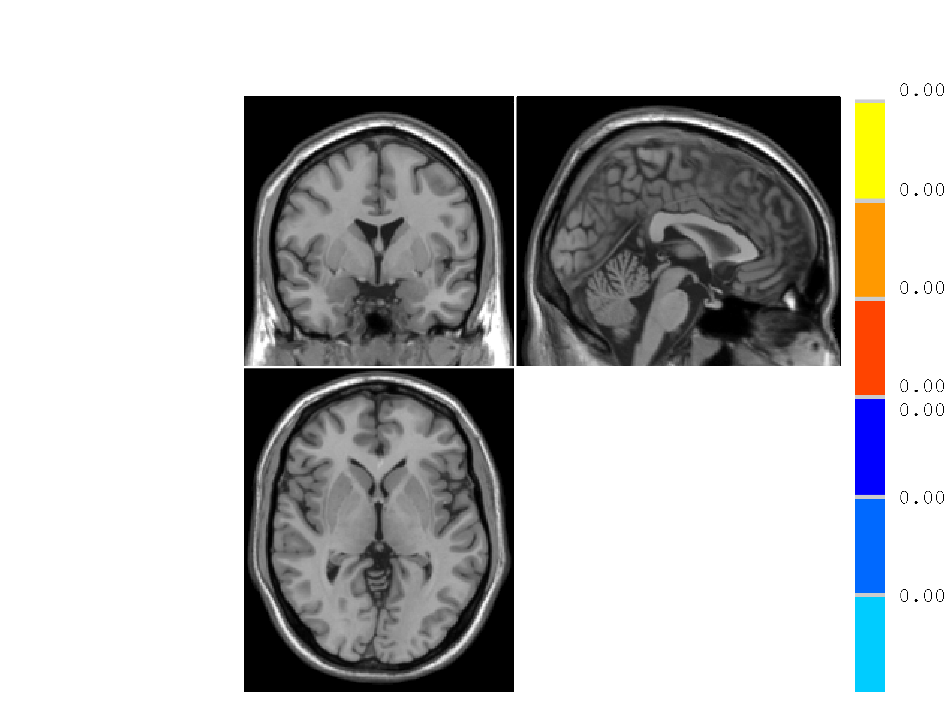


Fig2. Brain regions with abnormal fALFF in typical low-frequency range and at slow-3 band in NE. The results were corrected by GRF (voxel P < 0.05, cluster P< 0.05).


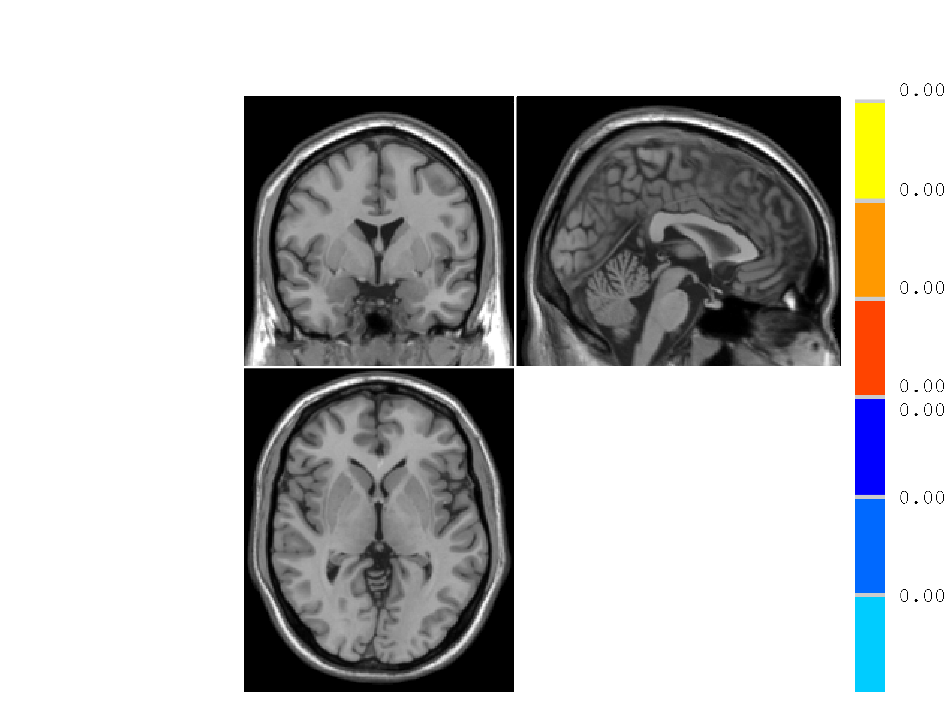


Fig3. Brain regions with abnormal fALFF in typical low-frequency range and at slow-4 band in NE. The results were corrected by GRF (voxel P < 0.05, cluster P< 0.05).


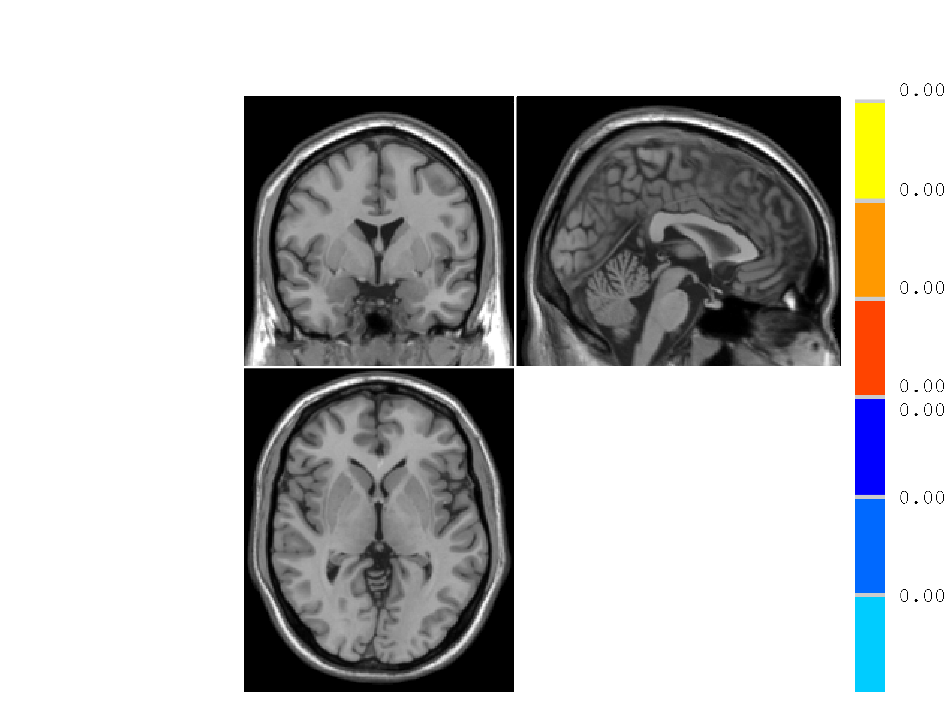


Fig4. Brain regions with abnormal fALFF in typical low-frequency range and at slow-6 band in NE. The results were corrected by GRF (voxel P < 0.05, cluster P< 0.05).


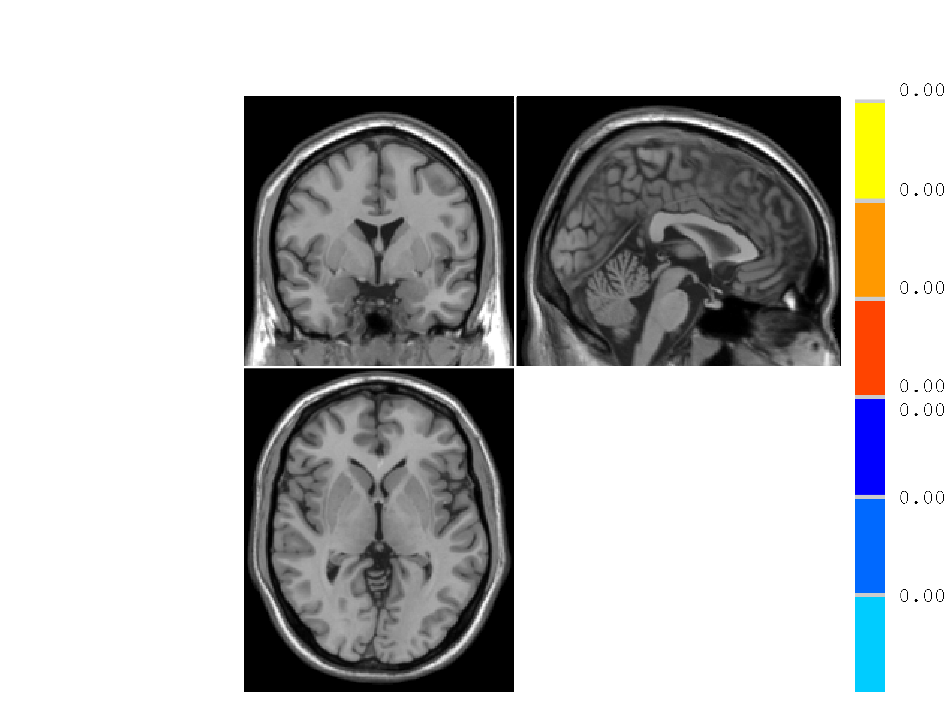


Fig5. Brain regions with abnormal PerAF in typical low-frequency range and at slow-2 band in NE. The results were corrected by GRF (voxel P < 0.05, cluster P< 0.05).


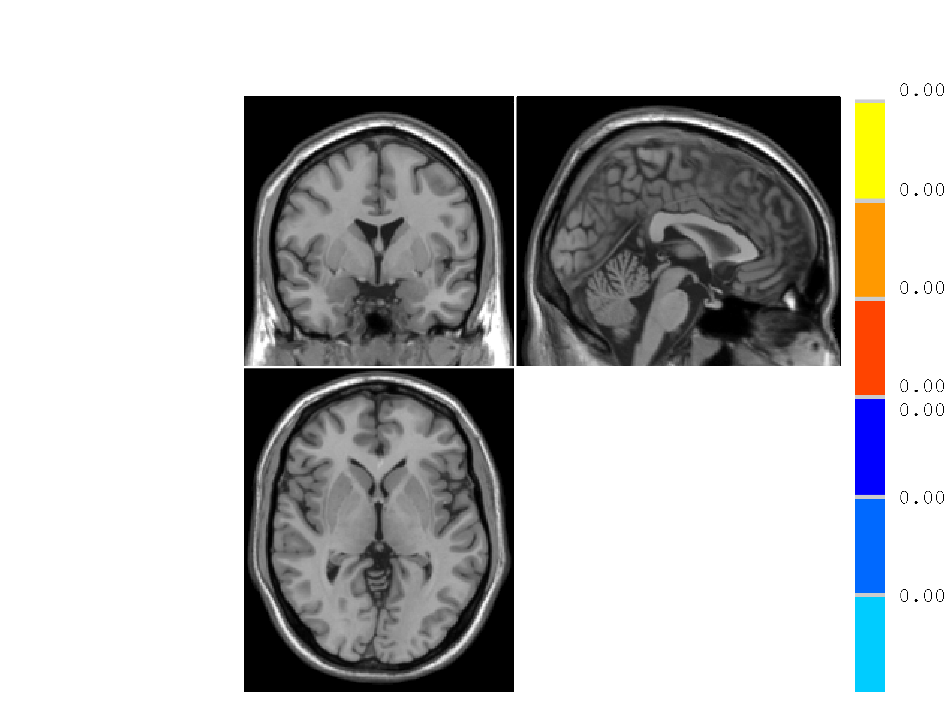


Fig6. Brain regions with abnormal PerAF in typical low-frequency range and at slow-3 band in NE. The results were corrected by GRF (voxel P < 0.05, cluster P< 0.05).


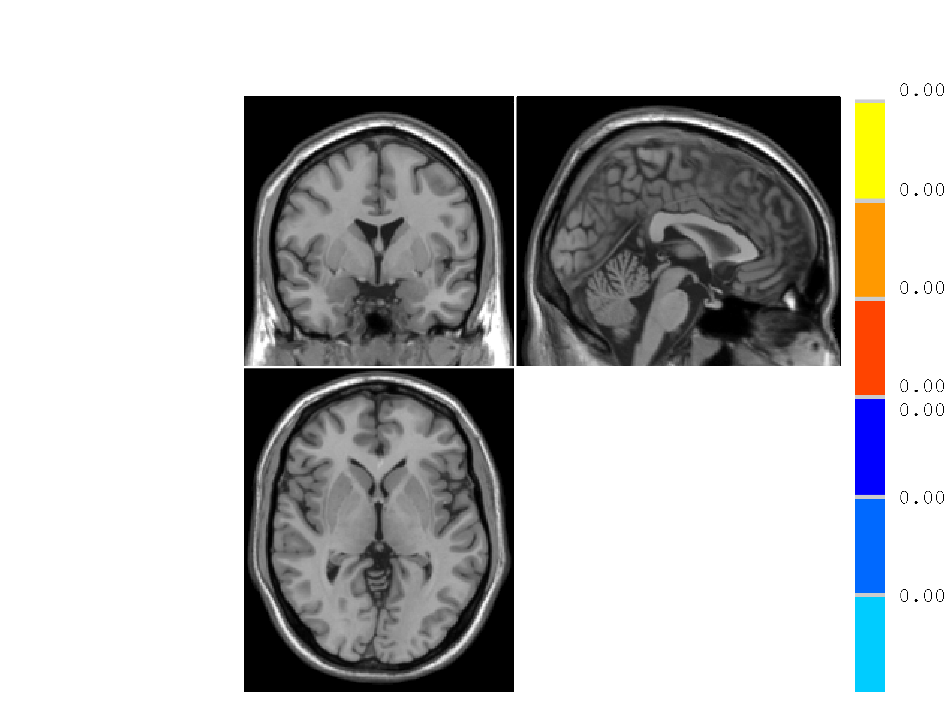


Fig7. Brain regions with abnormal PerAF in typical low-frequency range and at slow-4 band in NE. The results were corrected by GRF (voxel P < 0.05, cluster P< 0.05).


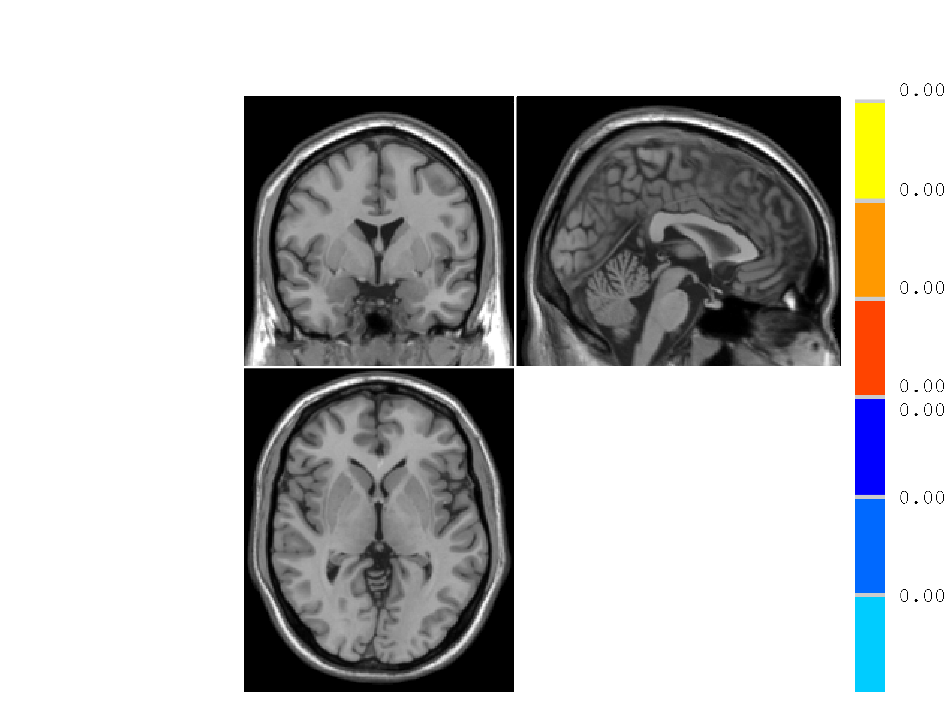


Fig8. Brain regions with abnormal PerAF in typical low-frequency range and at slow-6 band in NE. The results were corrected by GRF (voxel P < 0.05, cluster P< 0.05).


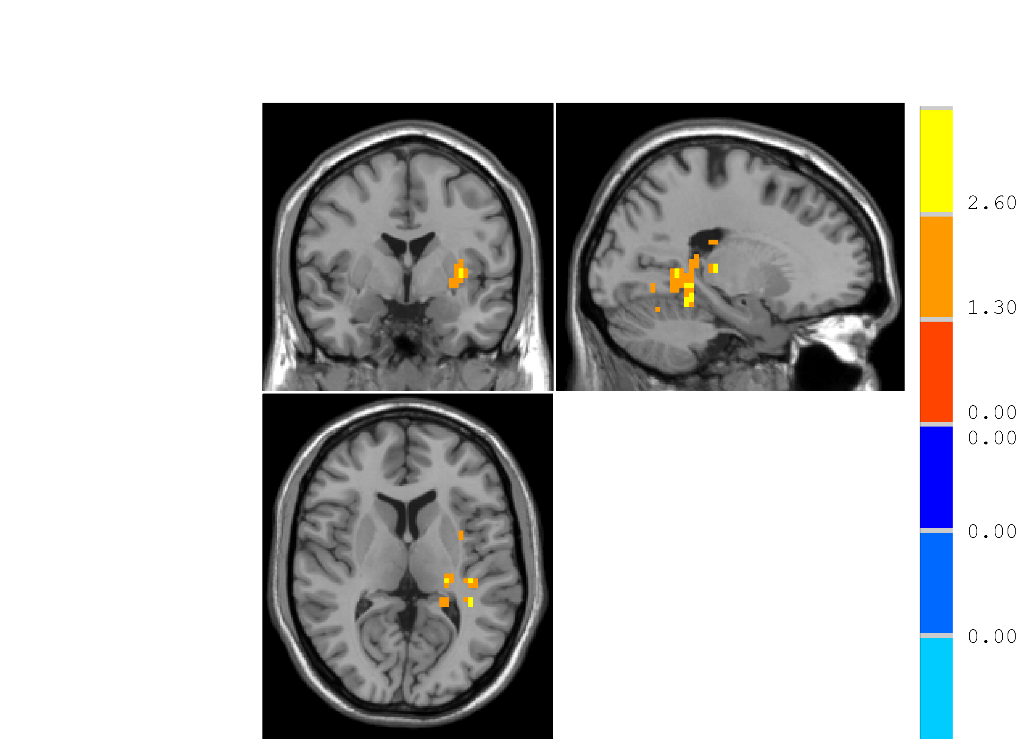


Fig9. Brain regions with abnormal ReHo in typical low-frequency range and at slow-2 band in NE. The results were corrected by GRF (voxel P < 0.05, cluster P< 0.05).


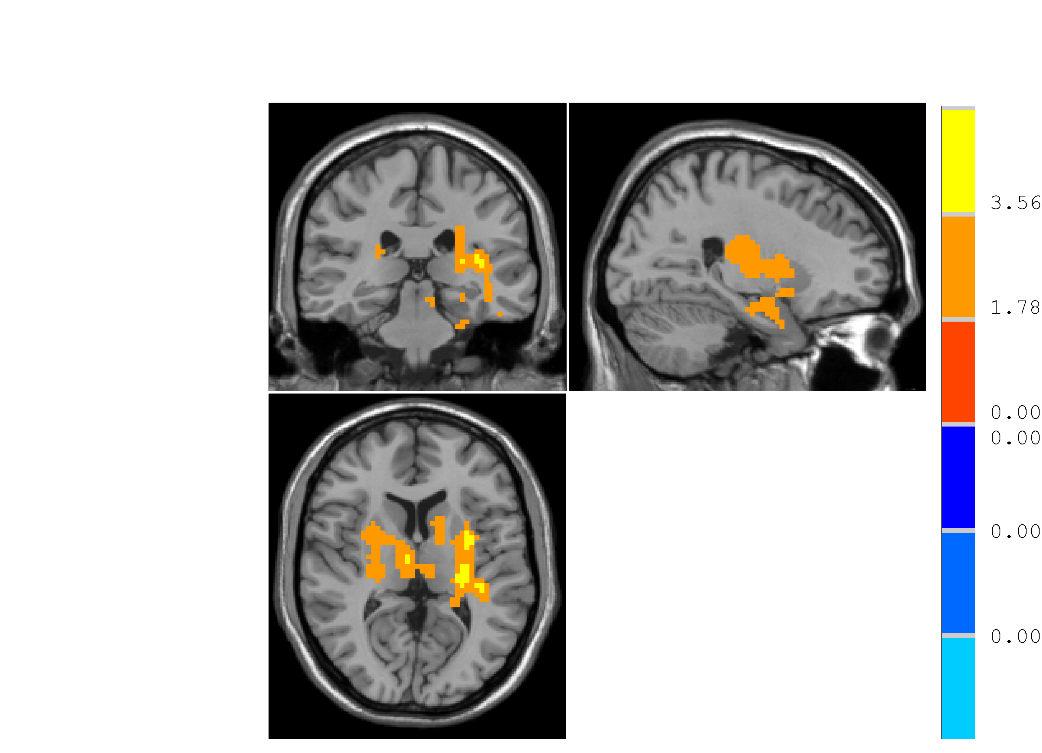


Fig10. Brain regions with abnormal ReHo in typical low-frequency range and at slow-3 band in NE. The results were corrected by GRF (voxel P < 0.05, cluster P< 0.05).


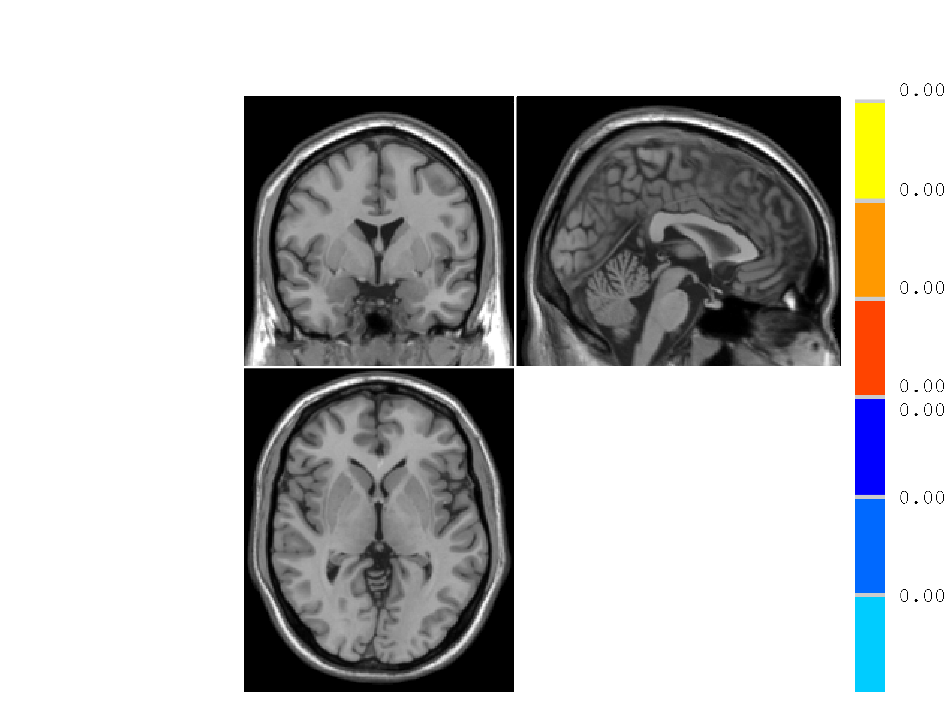


Fig11. Brain regions with abnormal ReHo in typical low-frequency range and at slow-4 band in NE. The results were corrected by GRF (voxel P < 0.05, cluster P< 0.05).


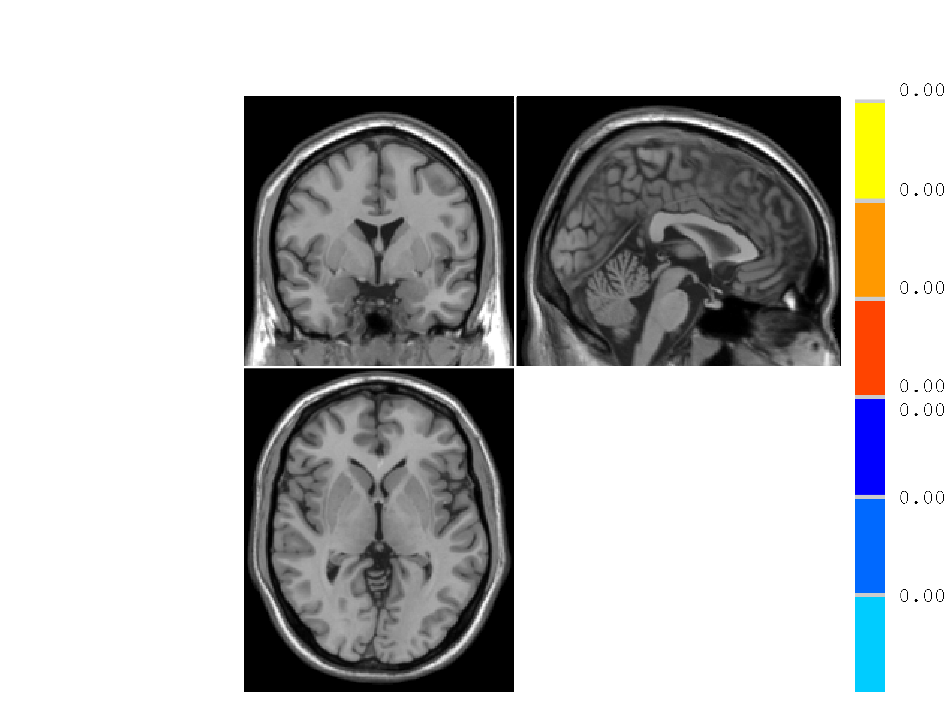


Fig12. Brain regions with abnormal ReHo in typical low-frequency range and at slow-6 band in NE. The results were corrected by GRF (voxel P < 0.05, cluster P< 0.05).
